# The Army Surgeon: His Status and Duties

**Published:** 1863-10

**Authors:** 


### THE ARMY SURGEON: HIS STATUS AND DUTIES.

I. The *rank* in the medical corps is like that in other corps, extending from that held by the Surgeon-General, which is that of a Brigadier General, to that held by the youngest (by appointment) Assistant-Surgeon, which is that of a 1st Lieutenant. A medical officer has choice of quarters according to his rank, and is entitled to the courtesies allowed his rank on all official occasions, as on court-martial, &c.

II. *Authority.*—The medical officer commands *in his own department;* his authority extending over junior medical officers, hospital attendants and inmates. He may order soldiers into or out of the hospital, even against the wishes of a ranking commanding officer; but is himself subject to arrest and trial by court-martial for the real or supposed improper use of this authority. In the absence of other commissioned officers he may command a post, or lead men in battle. In the presence of other commissioned officers, he does not command out of his own department, even though such commanding officer ranks only as Lieutenant, whilst the Surgeon ranks as Major.

III. His *duties* are, 1st. To *request* of his immediate commanding officer that quarters, attendants, and transportation be furnished for the sick; 2nd. To provide medical and hospital supplies to meet the necessities of the command; 3d. To attend to the sick, using all necessary oversight to insure proper attention on the part of the attendants; 4th. To bring to the notice of his immediate commander any and every fact bearing upon the health of the command, in order that the proper action may be taken and the appropriate orders issued to the Quartermaster, Commissary, or others. In this way only can he *authoritatively* influence the camp police, ventilation of quarters, the mode of cooking, hours of eating, &c.; but he is not debarred from using the powers of advice and persuasion, often more potential than military orders in these matters.—*Sanitary Reporter.*

